# Symptomatic postoperative discal pseudocyst following percutaneous endoscopic lumbar discectomy: A case report and review of the literature

**DOI:** 10.1097/MD.0000000000024026

**Published:** 2021-01-22

**Authors:** Junjie Li, Shuhan Liang, Wei Xie, Jinxin Luo, Jin Tang, Liu Liu, Ying Li, Congjun Wu, Xugui Li

**Affiliations:** a Hubei 672 Orthopaedics Hospital of Integrated Chinese & Western Medicine, Wuhan, 430079, Hubei; b Hunan Liuyang Orthopaedics Hospital, Liuyang, 410327, Hunan, China.

**Keywords:** interventional therapy, lumbar disc herniation, percutaneous endoscopic lumbar discectomy, postoperative discal pseudocyst

## Abstract

**Rationale:**

Percutaneous endoscopic lumbar discectomy (PELD) is an effective treatment for lumbar disc herniation and postoperative discal pseudocyst (PDP) can rarely develop after PELD.

**Patient concerns:**

A 30-year-old man experienced low back pain and pain in the right lower extremity for 1 month, which aggravated for 3 days.

**Diagnoses:**

Preoperative CT and MRI showed lumbar disc herniation at the L4/5 level. Then the patient underwent PELD under local anesthesia and his symptoms disappeared immediately after surgery. After 37 days of PELD, the patient complained of recurrent low back pain on the right side, and pain on the outer side of his lower leg. MR imaging revealed cystic mass with low signal on T1-weighted images (T1WI), and high signal on T2-weighted images (T2WI). The patient was diagnosed with a symptomatic PDP after PELD.

**Interventions:**

Initially, the patient was treated with conservative treatment, including administration of aescin and mannitol by intravenous infusion, physical therapy, sacral canal injection. Then he underwent discography at L4/5 and ozone ablation under local anesthesia.

**Outcomes:**

The patient's condition improved significantly after 1 week of surgery and was discharged. One-year and 3-month follow-up revealed no recurrence of low back pain and leg pain.

**Lessons:**

PDP is one of the rare complications of PELD, usually occurs in young patients. Patients with PDP have a low signal intensity on T1WI and high signal intensity on T2WI, which can be treated by conservative treatment, interventional therapy, and surgical treatment.

## 1 Introduction

At present, percutaneous endoscopic lumbar discectomy (PELD) is an effective surgical method for the treatment of lumbar disc herniation, and has the advantage of less surgical trauma, fewer complications, and lower risk of postoperative recurrence. But, there are still some risks and complications associated with PELD. Kang and Park^[1]^ first reported postoperative discal pseudocyst (PDP) after PELD in 2011. Thereafter, few cases of PDP have been described in the literature, most studies were case reports. Herein, we present a case who developed PDF after PELD.

## 2 Case presentation

A 30-year-old man presented with low back pain and pain in the right lower extremity for 1 month, which aggravated for 3 days. After being admitted to our hospital, the patient could not walk upright due to severe pain. He was previously healthy and denied the history of hypertension and diabetes.

**Figure 1:**
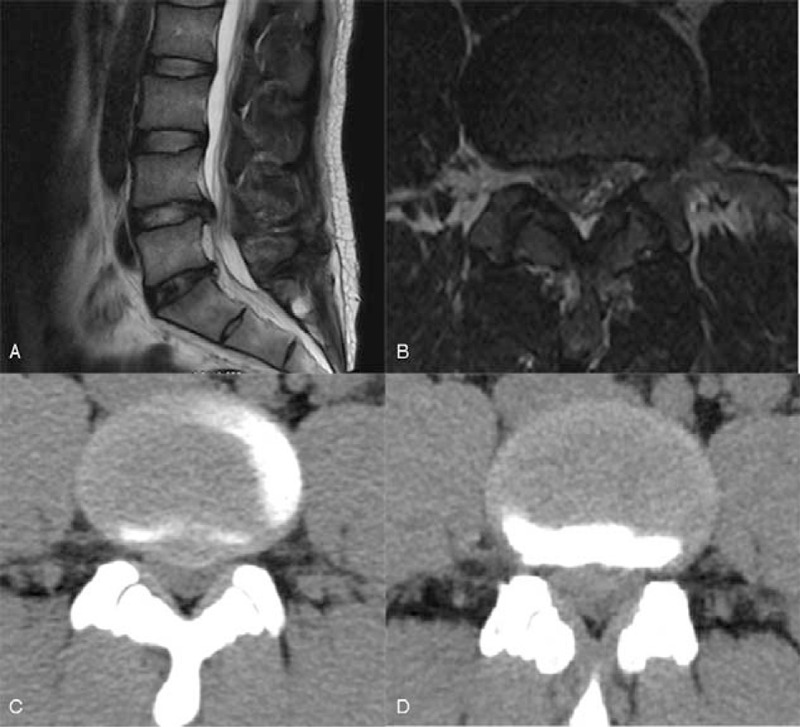
Preoperative CT and MR imaging showed central to right sided disc herniation at the L4/5 level.

**Figure 2:**
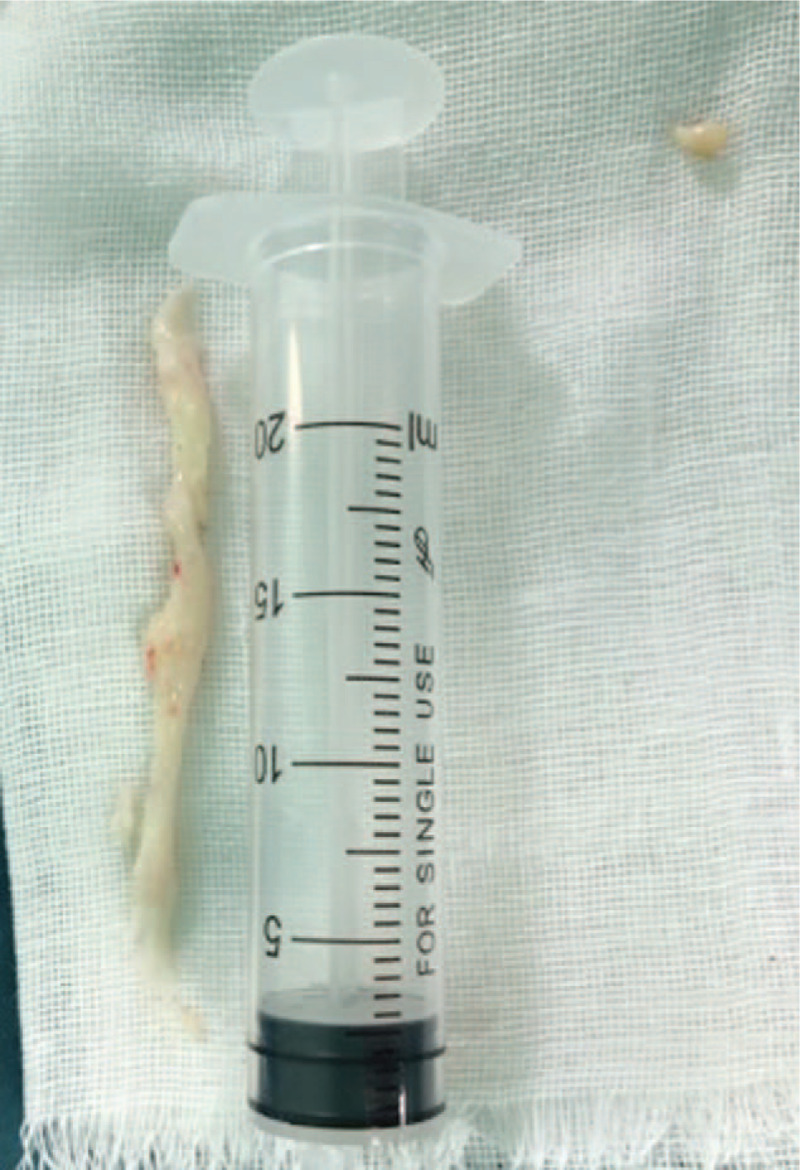
Nucleus pulposus tissue removed during the surgery had high water content, rich in type II collagen and was highly viscous.

On physical examination, patient had pain upon pressure on L4/5 interspinous tissue and right paraspinous region, range of motion of the hip joint was normal, straight-leg raising test and augmentation test were 30 degree on the right (positive) and 70 degree on the left (negative), right knee and right ankle reflexes were weakened. Strength in extensor hallucis longus of the right foot was grade 4, and no abnormalities were found in other muscles of the lower extremities. The bilateral femoral nerve stretch test was negative. There was no obvious abnormal skin sensation in the perineal and sellar regions. Bilateral Babinski's signs were negative, Visual Analogue Scale (VAS) score was 9 points. Preoperative CT and MRI of the patients showed central to right sided disc herniation at the L4/5 level (Fig. 1). There were no absolute contraindications for PELD. The patient underwent PELD for L4/5 disc herniation under local anesthesia, and the nucleus pulposus tissue removed during the surgery is shown in Figure 2. The low back pain and pain in the right lower extremity of the patient disappeared immediately after surgery with a VAS score of 1. Patient was given routine medications after surgery. After 24 hours of surgery, the patient got out of bed and walked under the protection of lumbar support belt. One week after the surgery, the condition of patient was obviously improved and he was discharged. The patient was asked to continue the functional exercise properly and not to perform heavy labor.

**Figure 3:**
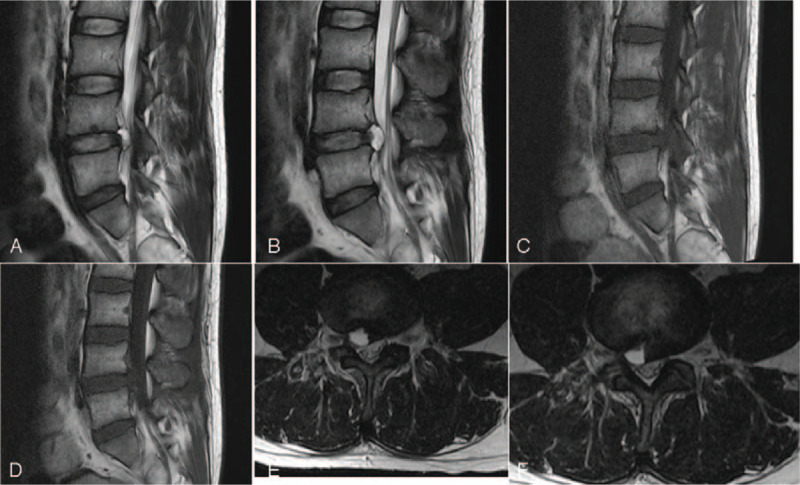
MR imaging at 1 mo after surgery. (A and B) Sagittal T2WI revealed cystic mass with high signal at L4/5 disc space; (C and D) sagittal T1WI revealed cystic mass with low signal at L4/5 disc space; (E and F) cross-sectional scanning revealed cystic mass with high signal on the right side of the central spinal canal at L4/5 disc space.

Thirty-seven days after the surgery, the patient complained of recurrent low back pain on the right side, and pain on the outer side of his lower leg, which aggravated when walking and seriously influenced the activities of daily life. The patient was then admitted to our hospital again. On physical examination, patient had slight tenderness upon pressure on L4/S1 interspinous tissue and right paraspinous region, hip joint range of motion was normal, straight-leg raising test and augmentation test were 50 degree on the right (positive) and 70 degree on the left (negative). Right knee and right ankle reflexes were normal. There was no obvious abnormality in the strength of muscles from lower extremities. The bilateral femoral nerve stretch test was negative. There was no obvious abnormal skin sensation in the perineal and sellar regions. Bilateral Babinski's signs were negative, VAS score was 8 points. MR imaging showed cystic mass with low signal on T1-weighted images (T1WI), and high signal on T2-weighted images (T2WI) (Fig. 3). Blood routine test upon admission showed white blood cells of 6.88 × 10^9^/L, neutrophil percentage of 54.5%; erythrocyte sedimentation rate of 7 mm/hour, and C-reactive protein of 0.2 mg/dL. After reviewing the literature, and considering the above clinical characteristics and imaging findings of the patient, the patient was diagnosed with symptomatic PDP after PELD.

**Figure 4:**
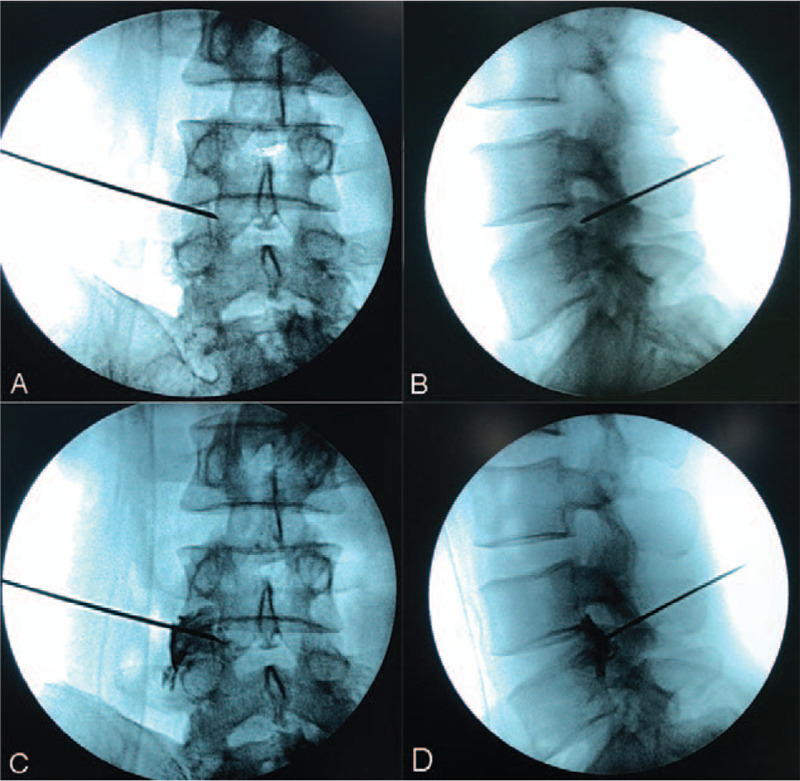
The position of puncture needle tip reached the target cystic area (A and B). Discography showed that the contrast media was filled within the local cystic mass, which was connected closely to the nerve root (C and D).

The patient was initially treated with conservative treatment, including administration of aescin and mannitol by intravenous infusion, physical therapy, sacral canal injection. After nearly 1 week of systemic treatment, patient's symptoms were not relieved. Then discography at L4/5 and ozone ablation was performed under local anesthesia. Results of intra-operative discography are shown in Figure 4. The low back pain and pain in the lower extremity disappeared immediately after the surgery, with a VAS score of 1 point. Patient was given routine medications after surgery, and after 24 hours of surgery, the patient got out of bed and walked under the protection of lumbar support belt. The patient's condition improved significantly after 1 week of surgery and discharged. The patient was asked to continue their functional exercise properly and not to perform heavy labor.

**Figure 5:**
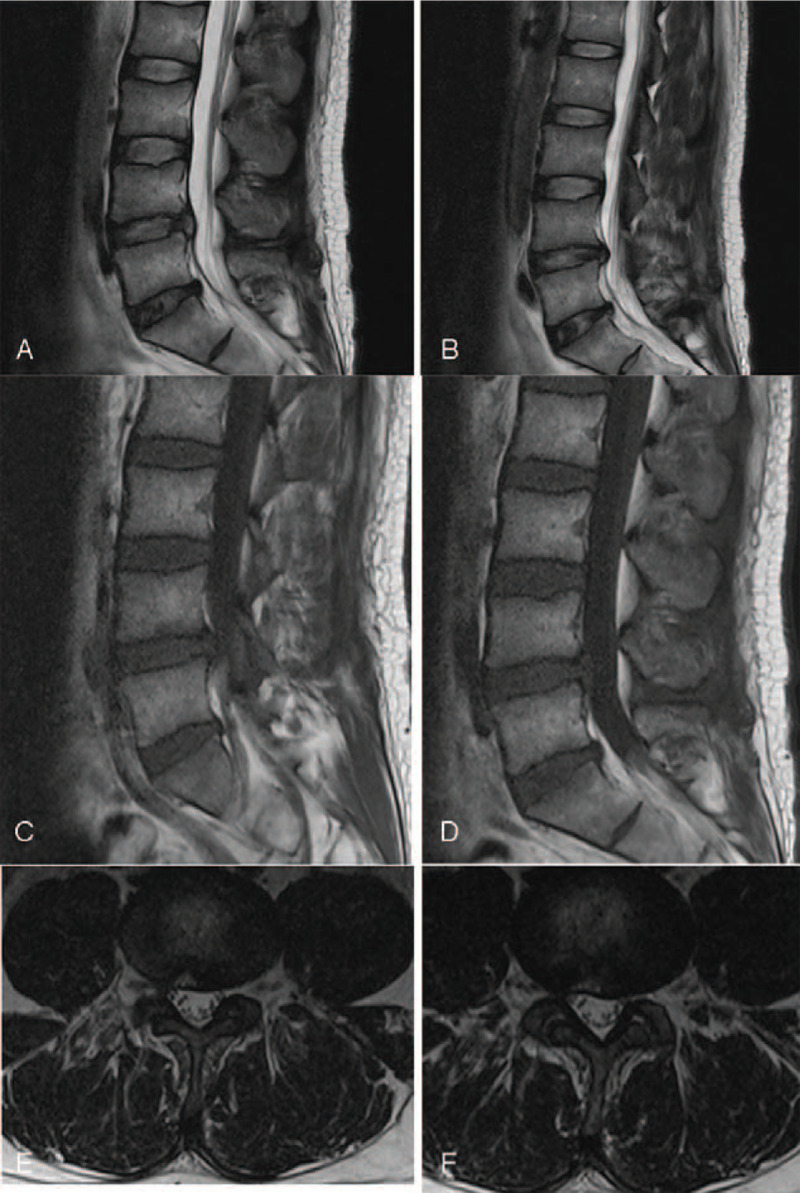
MR imaging at 3 mo after surgery revealed the disappearance of cystic mass that was found originally at L4/5 level on the right side, the cystic long T2 signal at the posterior part of the L4–5 disc, and also the herniated disk at the level of L4/5. (A and B) Sagittal T2WI; (C and D) sagittal T1WI; (E and F) cross-sectional scanning.

The patient had no complaints of low back pain and leg pain (VAS score was 0 points) at 3 months of follow-up. MR imaging showed that cystic mass that was found originally at L4/5 level on the right side had disappeared; the cystic long T2 signal at the posterior part of the L4–5 disc had disappeared, and L4/5 disc herniation had also disappeared (Fig. 5). The patient had no complaints of low back pain and leg pain (VAS score was 0 points) at 1-year follow-up (MRI examination was not performed).

## 3 Discussion

Discal cyst was first reported in 2001 by Chiba et al.^[2]^ Discal cysts are rare intraspinal lesions, presenting with symptoms similar to those caused by lumbar disc herniation, and hence are often misdiagnosed. At present, patients with discal cyst are rarely reported; relevant studies are mostly case reports. Discal cyst most commonly occurs in the lumbar spine. The diagnosis of discal cysts is mainly based on MRI. The typical MRI findings include the presence of cystic mass with connections to an intervertebral disc, and low signal intensity on T1WI and high signal intensity on T2WI. Chiba^[2]^ and Lee^[3]^ summarized the characteristics of discal cysts:

1) the clinical symptoms of discal cysts are similar to those of typical lumbar disc herniation, manifesting as a unilateral single nerve root lesion;
2) the age of the patients with discal cysts are younger than those with typical lumbar disc herniation;
3) MRI findings show round or oval cysts with low and high signal intensities on T1- and T2-weighted images, respectively;
4) CT or MRI shows the minimal degeneration of the involved disc;
5) discography shows connections between the cysts and the corresponding intervertebral disc in the lumbar spine, and patients experience radiating pain in the lower extremity after injection of the contrast media;
6) the symptoms are relieved immediately after the cyst is removed;
7) the cyst wall consists of dense fibrous connective tissue, containing bloody to clear liquid;
8) histological examination shows no intervertebral disc material inside the cyst.

However, the mechanisms of formation of discal cysts are still unclear. At present, two hypotheses for the formation of discal cysts have been suggested.^[4–6]^ Toyama et al^[7]^ reported 7 cases with discal cysts, and found bloody serous fluid inside the cysts in 4 cases. It is considered that hematoma occurs due to tearing of the epidural venous plexus, and reabsorption of hematoma causes the formation of discal cysts. Kono^[6]^ proposed that the pathogenesis of discal cysts is similar to that of meniscal cysts. They hypothesized that discal cysts are caused by focal inflammation, example, after disc herniation, the nucleus pulposus flows out of the disc, which causes an inflammatory response, and eventually leads to reactive pseudomembrane formation and the development of discal cysts. Demaerel et al^[8]^ proposed that the discal cyst is related to the collagen connective tissue in the intervertebral disc. After intervertebral disc degeneration, collagen connective tissue in the intervertebral disc softens and liquidates, and in a few cases, a pseudomembrane can be formed to encapsulate the liquid, forming a cyst.

In the present study, the patient experienced low back pain and radicular pain in the right lower extremity after 37 days of PELD. Before the MRI examination, we speculated that the patient might have recurrent disc herniation in the same segment. However, MRI of the lumbar spine showed a cystic mass formed at the L4/5 intervertebral disc levels on the right side, which was connected with the annulus fibrosus, and caused mechanical compression of the right L5 nerve root. MRI showed slightly mixed signals within the mass and low signal on T2-weighted image and high signal on T1-weighted image, which is similar to the signal intensities of the discal cysts. On literature review, we found that in 2011, Kang and Park^[1]^ reported the development of symptomatic PDP after PELD. They observed that among 1503 patients treated with PELD, symptomatic PDP was found on postoperative follow-up MRI in 15 patients, with an incidence of 1.0%, the mean age of those patients was 22.5 years, the mean postoperative interval from surgery to PDP detection was 53.7 days. Liu et al^[9]^ reported a patient with lumbar spinal stenosis and lumbar disc herniation who developed PDP after treatment with open fusion and internal fixation. Based on the literature reports and the clinical characteristics and imaging findings, the patient in the present study was diagnosed with symptomatic PDP after PELD. PDP has similar characteristics to discal cysts.^[10]^ The pathogenetic mechanisms of PDP are also uncertain. Young^[11]^ proposed that after the discectomy, a small amount of residual free disc tissue debris at the original site of disc herniation changed into inflammatory granulation tissues through absorption and inflammatory hyperplasia. The inflammatory granulation tissue formed a pseudocyst, and the disc tissue debris is encapsulated by the pseudocyst, and the cyst wall is connected with the intervertebral disc. If the intervertebral disc tissue debris is completely absorbed, and the cyst cavity is not destroyed, the oozing liquid can accumulate in the cyst cavity, therefore the pressure within the cyst cavity is gradually increased, which can cause mechanical compression of the nerve similar to the compression caused by disc herniation.

After searching Pubmed and China National Knowledge Infrastructure (CNKI), we found 6 studies (30 cases) reporting the development of PDP after lumbar spine surgery. In one of the 6 studies, only 15 of 4503 LDH patients developed symptomatic PDP on follow-up MRI after PELD. And we encountered only 1 patient with symptomatic PDP among nearly 2000 patients with lumbar disc disease who underwent PELD in our department, indicating that the incidence rate of PDF is low. The mean age of those patients reported in the literature was 25.3 years. The mean postoperative interval from surgery to PDP detection was 40.3 days. One of those 32 cases developed PDP after open surgery, and the remaining 31 patients developed PDP after microendoscopic diskectomy (MED) and percutaneous endoscopic discectomy (PED). Eighteen patients were treated conservatively, 11 patients were treated with MED and PED again, 1 patient underwent lumbar laminectomy and 1 patient underwent puncture and aspiration, and the 31 patients had good prognosis. The treatment option and prognosis of 1 patient with PDP after open surgery was not reported.

Treatment options for patients with PDP include conservative treatment, interventional, and surgical therapies:

1) Conservative treatment: Subash^[12]^ reported that 2 patients under the age of 20 experienced low back pain and leg pain after MED. After MRI, the patients were diagnosed with PDP. Because the symptoms could be tolerated, conservative treatment was adopted in the 2 patients. After 3 months of follow-up, MRI showed narrowing of the pseudocysts. At 6 months of follow-up, MRI showed that the area of the cyst disappeared, and the low back pain and leg pain was also significantly improved.
2) Interventional therapy: The use of interventional therapy in treating PDP has not been reported. But interventional therapy has been used to treat discal cysts. Yu et al^[13]^ performed aspiration and steroid injection under the guidance of C-arm in a 27-year-old patient with discal cysts. After 1-week, 1-month, and 3-month follow-up the patient had no abnormal neurological symptoms and no persistent low back and leg pain. Yoshimi et al^[14]^ also treated a 51-year-old patient with discal cysts under CT guidance and the patients also obtained good outcomes. Therefore, interventional therapy under the guidance of DR or CT can also be able used to treat PDP. In the present study, we performed targeted puncture and ozone injection for the treatment of PDP. Targeted puncture of the pseudocyst was performed at level L4/5 under the guidance of DR. The pseudocyst was treated successfully, the patient's symptoms disappeared immediately after surgery, and the patient had a good prognosis.
3) Surgical therapy: Different surgical methods are used to treat PDP. Hiroaki et al^[15]^ used percutaneous endoscopic discectomy to treat a patient who developed PDP after receiving PELD at L4–5, and the short- and long-term outcomes were good. Some authors have also used MED to treat PDP. Ryutaro et al^[16]^ encountered 2 patients with PDP after PELD. One patient was treated with MED and the other patient was treated with PELD again. The symptoms of the 2 patients were improved immediately after treatment. Intra-operatively the author observed spouting serous fluid and tissues of cyst wall spouted after piercing the cyst wall. The patients’ low back pain and leg pain disappeared during the follow-up period.

Kang reported 15 patients with symptomatic PDP, whose mean age was 22.5 years. In this present study, the patient was 30 years old. The nucleus pulposus tissue removed during surgery consisted mainly of water and collagen, which had high viscosity. Therefore our conjecture on the mechanism of PDP development is similar to the pathogenetic mechanisms of PDP proposed by Young et al.^[11]^ In young individuals, the nucleus pulposus tissue has more water content, and is rich in type II collagen. After surgery for disc herniation, local nucleus pulposus debris has high type II collagen content and becomes highly viscous, which promotes the formation of the cyst wall of the PDP to a certain extent. But in middle-aged people, nucleus pulposus tissue has less water content, high degree of degeneration, and low II collagen content. So it is difficult to come across PDP in middle-aged and older patients. In the present study, we used interventional therapy under the guidance of DR to treat the patient with symptomatic PDP, which showed good outcomes at 3-month follow-up. The use of interventional therapy in the treatment of PDP has not been reported in the literature. The disadvantage of interventional therapy is that we cannot clearly see the shape and contents of pseudocysts, their relationship with intervertebral discs and nerves, and cannot obtain tissue specimens for histological examination.

Symptomatic PDP is a rare complication following PELD that usually occurs in young patients. The mechanism underlying the occurrence of PDP is not completely clear, which may be related to various causes. PDP causes symptoms (including low back and leg pain) similar to those caused by disc herniation. However, PDP can be differentially diagnosed from disc herniation by MRI examination. Patients with PDP can be treated by conservative treatment, interventional therapy, and surgical treatment. With the increased incidence of spinal diseases, patients who received surgical treatment will increase, and the possibility of symptomatic PDP after surgery will also increase. We should pay more attention to this disease, study its underlying mechanism, optimize treatment options, and reduce the development of such complications.

## Author contributions

L.J.J. and L.S.H wrote the paper. X.W, L.J.X and T.J consulted the literature and examined the articles. L.L, L.Y, W.C.J performed the operation surgery. L.X.G developed the idea for the study.
